# Hemodynamic Characterization of Primary Hypertension in Children and Adolescents

**DOI:** 10.1161/JAHA.119.015097

**Published:** 2020-06-06

**Authors:** Ye Li, Haotian Gu, Manish D. Sinha, Phil Chowienczyk

**Affiliations:** ^1^ King's College London British Heart Foundation Centre London United Kingdom; ^2^ Department of Pediatric Nephrology Evelina London Children's Hospital London United Kingdom

**Keywords:** arterial pressure, cardiac output, cardiovascular disease, hemodynamics, primary hypertension, Hypertension, High Blood Pressure

## Abstract

**Background:**

Primary hypertension in children is often characterized by high pulse pressure that could be attributable to increased ventricular ejection velocities and volumes and/or increased arterial stiffness. The objective of this study was to examine the contributions of cardiac (ventricular ejection) and vascular (systemic vascular resistance, arterial stiffness, and pressure wave reflection) properties to primary hypertension in children and adolescents.

**Methods and Results:**

Children aged 8 to 18 years referred to a tertiary center for evaluation of hypertension and found to have primary hypertension (n=31) were compared with normotensive controls of similar age (n=50). Peripheral (brachial) and central (carotid) blood pressure waveforms and carotid‐femoral pulse wave velocity were measured by tonometry. Left ventricular outflow tract velocities and ejection volumes were measured by echocardiography. Wave separation and wave intensity analysis were used to assess pressure wave propagation. Increased mean arterial pressure in hypertensive children (90±15 versus 76±10 mmHg in hypertensive versus normotensive children; means±SD;* P*<0.001) was explained by increased heart rate and cardiac output (5.3±2.0 versus 4.5±1.2 L/min adjusted for age and sex; *P*<0.05) rather than increased systemic vascular resistance (18.0±4.6 versus 19.3±7.3 mmHg/min/mL; *P*=0.374). A more‐marked increase in pulsatility (peripheral pulse pressure 66±21 versus 46±12 mmHg; *P*<0.001) was explained by increased proximal aortic stiffness (pulse wave velocity, 3.3±1.4 versus 2.5±0.8 m/s; *P*<0.005) and increased left ventricular ejection velocity (1.33±0.24 versus 1.21±0.18 m/s; *P*<0.05).

**Conclusions:**

Cardiac overactivity characterized by increased heart rate and left ventricular ejection velocities and increased proximal pulse wave velocity may be the main cause of primary hypertension in children.

**Clinical Perspective**

**What Is New?**

- Hypertension in children with primary hypertension is predominantly attributed to overactivity of the heart (increased heart rate and increased ejection velocities) and increased proximal aortic stiffening.

**What Are the Clinical Implications?**

- Given the tracking of hypertension from children to adults, the finding of a cardiac/aortic rather than peripheral vascular cause of primary hypertension has implications for the etiology of hypertension both in children and adults.
- It also has implications for the best treatment in children: Clinical trials of interventions designed to reduce cardiac overactivity should be considered.

Hypertension is one of the most important global public health problems, but its etiology, particularly in children, is still poorly understood. In children and young adults, hypertension often takes the form of isolated systolic hypertension with a wide pulse pressure.^1^ High pulse pressure is also observed in old age, where it has been attributed to stiffening of large elastic arteries limiting the cushioning of blood ejected by the left ventricle by the arterial tree and possibly other hemodynamic effects.^2^ Arterial stiffening is well recognized as part of an age‐related degenerative process.^3^ In children, a degenerative process seems unlikely, but other mechanisms of arterial stiffening could occur and there may be other causes of increased pulse pressure, such as an increase in ventricular ejection or increased pressure wave reflection.

The aim of the present study was to characterize central arterial hemodynamics and investigate the mechanisms of primary hypertension in children and adolescents, specifically to examine the contributions of cardiac (left ventricular [LV] ejection velocities and volumes) and vascular (systemic vascular resistance, arterial stiffness, and pressure wave reflection) properties to hypertension.

## Methods

### Study Population

Data used in this study will be made available to any researcher for the purposes of reproducing the results reported or use in other studies.

Children and adolescents (subsequently referred to as “children”) up to age 18 with primary hypertension referred to the pediatric hypertension clinic of the Evelina London Children's Hospital (London, UK) were consecutively recruited to a prospective observational study investigating the relationship of target organ damage to blood pressure (BP). Age‐matched healthy children were recruited contemporaneously from the local community. Hypertension was defined as average systolic or diastolic BP ≥95th percentile (equivalent to z‐score ≥1.645) or if the patient was on antihypertensive therapy, according to definitions of hypertension in the fourth report on the diagnosis, evaluation, and treatment of high BP in children and adolescents.^4^ Children with chronic kidney disease or other causes of secondary hypertension were excluded from the study. Additional exclusion criteria included those with congenital heart disease, cardiac arrhythmias, and inability to obtain high‐quality cardiovascular measurements. The institutional ethics committee approved the study, children gave their assent, and all parents gave written informed consent. Anthropometric, clinical, and laboratory data were collected on the day of the research investigations. Healthy children underwent all examinations except for venesection.

### BP, Arterial Tonometry, and Pulse Wave Velocity

Hemodynamic measurements were made with children in a supine position in a quiet environment. Peripheral systolic and diastolic BP were measured in triplicate by a trained observer by auscultation, using a calibrated aneroid sphygmomanometer and an appropriate‐size arm cuff according to the British and Irish Hypertension Society guidelines. Given the age‐related change in BP throughout childhood, all peripheral BP measurements were expressed as z‐scores (the number of SDs above or below a population mean assigned a value of zero).^4^ Radial and carotid pressure waveforms were obtained by applanation tonometry performed by an experienced operator using the SphygmoCor system (AtCor Medical Pty Ltd, Sydney, NSW, Australia). Approximately 10 cardiac cycles were ensemble averaged. Waveforms that did not meet the in‐built quality‐control criteria in the SphygmoCor system were rejected. Peripheral systolic and diastolic BP were used to calibrate radial waveforms and thus to obtain a mean arterial pressure (MAP) through integration of the radial waveform. Carotid waveforms were calibrated from MAP and diastolic brachial BPs on the assumption of the equality of these pressures at central and peripheral sites.^5^ Carotid‐femoral pulse wave velocity (PWV) was calculated from sequential recordings of the carotid and femoral artery pressure waveforms using the same SphygmoCor device and transducer. The difference in time of pulse arrival between the 2 sites referenced to the R‐wave of the ECG was taken as the transit time. The path length between these 2 sites was estimated from the distance between the sternal notch and femoral artery at the point of applanation and PWV calculated as the quotient of path length and transit time. All measurements were made in triplicate, and mean values were used for analysis.

### LV Outflow Tract Flow Velocity and Ejection Volumes

Ultrasound imaging was performed by an experienced operator using the Philips IE33 or Epiq ultrasound system (Philips Healthcare, Andover, MA). Flow velocity in the LV outflow tract (LVOT) was recorded using pulsed wave Doppler obtained from an apical 5‐chamber view. Flow velocity measurements were averaged over at least 3 cardiac cycles. Internal diameter of the LVOT was measured in the parasternal long‐axis view and LVOT area calculated assuming circularity. Direct measurements of time‐resolved LV volume, end‐diastolic volume and end‐systolic volume were also measured using the Philips QLab analysis package (Philips Healthcare) from 2‐dimensional apical views.

### Waveform Postprocessing

BP pressure waveforms obtained by tonometry and LVOT flow velocity waveforms were processed offline using custom software written in MATLAB (The MathWorks, Inc, Natick, MA) to characterize waveform morphology, perform forward and backward waveform separation, and hence determine reflection coefficients.

### BP Pressure Wave Morphology

**Figure 1:**
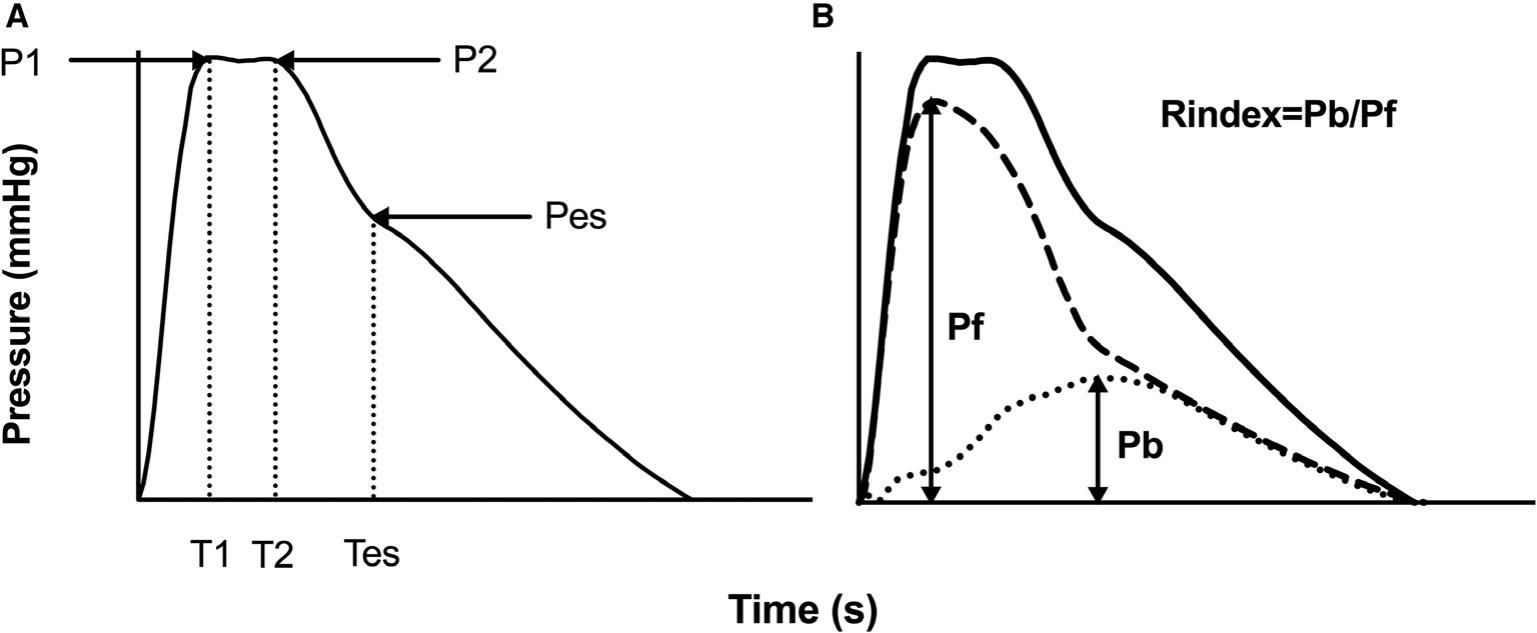
Central (carotid) pressure waveform. (**A**) Pressure at first systolic shoulder (P1) and second systolic shoulder (P2). (**B**) Forward (dashed line) and backward (dotted line) wave components that summate to equal total pressure (solid line). Reflection index (Rindex) is defined as the ratio of the magnitude of backward wave (Pb) to that of forward wave (Pf). Pulse pressures at P1, P2, and Pes are denoted by PP1, PP2, and PPes and can be divided into forward and backward components: PP1=PP1f+PP1b.

The first (P1) and second (P2) systolic shoulders/peaks and end‐systolic (Pes) points of the carotid pressure waveform, and their timings (T1, T2, and Tes) relative to the upstroke of the pressure waveform (Figure 1) were identified as the first and second local minima of the first derivative of the carotid pressure curve and confirmed by visual inspection by an observer blinded to the results. Pulsatile components of pressure at these points (PP1, PP2, and PPes) were obtained by subtracting diastolic BP from P1, P2, and Pes. Augmentation pressure was taken as the difference between P2 and P1. Augmentation index (AIx) was defined as augmentation pressure/central pulse pressure×100%.

### Flow and Volume Measurements

Aortic flow velocity was multiplied by the LVOT cross‐sectional area to obtain flow, which was integrated over time to obtain the stroke volume (SV_flow_) and cardiac output (CO) as the product of SV and heart rate (HR). SV was also obtained as the difference between LV end‐diastolic and end‐systolic volumes (SV_vol_). Ejection volumes (V1 and V2) corresponding to times T1 and T2 were obtained by integration of the aortic flow waveform from the start of systole to T1 and T2.

### Forward and Backward Pressure Wave Decomposition and Wave Intensity Analysis

Pressure wave decomposition was performed using Parker's time‐domain approach,^6^ based on conservation of mass and momentum, to obtain forward (P_f_) and backward (P_b_) pressure components of central pulse pressure so that: P_f_+P_b_=P–P_d_, where P is total pressure and P_d_ is the diastolic pressure. P_f_ and P_b_ are given by (Equations 1 and 2):

(1) $$P_{f}=\frac{1}{2}\sum \left[\left(dP+\rho \mathrm{cdU}\right)\right]$$

(2) $$P_{b}=\frac{1}{2}\sum \left[\left(dP-\rho \mathrm{cdU}\right)\right]$$

where U is flow velocity, ρ is blood density, and c is proximal aortic PWV, which was estimated using the method of the sum‐of‐squares (PWVss).^7^ Reflection index was defined as the ratio of amplitude of backward to that of forward wave.

Wave intensity, the flux of wave energy per unit area, was calculated as dI=dPdU and separated into forward and backward components (Equation 3):

(3) $$dI_{\pm }=\frac{1}{4\rho c}{(dP\pm \rho \mathrm{cdU})}^{2}$$

Wave intensity is positive for forward waves and negative for those that are traveling in a backward direction. Total wave energy can be obtained by integrating Equation 3 with respect to time.

### Determinants of Steady State and Pulsatile Components of BP

Contributions of cardiac and arterial properties to the steady‐state component of BP in hypertensive versus normotensive children were assessed by comparing the percentage difference between MAP in hypertensive and normotensive children with that of CO and systemic vascular resistance (SVR) using the relation MAP=CO×SVR. Cardiac and arterial contributions to greater PP1 (which, in most children, was equal to the central pulse pressure) in hypertensive versus normotensive children were assessed by comparing the difference in PP1 in hypertensive versus normotensive children with that of PWVss and U1, given that previous work has shown that, to a first approximation, for similar LVOT/proximal aortic geometry, PP1 is proportional to PWVss×U1.^8^

### Statistical Analysis

Subject characteristics are presented as means±SD. Comparison of hemodynamic variables between hypertensive and normotensive groups was made using 1‐way ANCOVA, with age and sex included as covariates. Results are presented as adjusted values (means±SE). Comparison of variables between untreated hypertensive and normotensive subjects was also performed. Analysis was performed using SPSS software (version 25; SPSS, Inc, Chicago, IL), and *P*<0.05 was taken as significant.

## Results

### Subject Characteristics

Table 1 shows the characteristics of children with hypertension (n=31) and normotensive controls of similar age (n=50). The proportion of male children was greater in the hypertensive compared with the normotensive group, and hypertensive children were taller and heavier, but with similar body mass index. Fifty‐eight percent of children with hypertension had isolated systolic hypertension and 42% systolic‐diastolic hypertension. Sixty‐four percent were taking antihypertensive medication.

**Table 1:** Characteristics of Normotensive and Hypertensive Children

Characteristic^a^	Normotensive (n=50)	Hypertensive (n=31)^b^	*P* Value
Age, y	14±3	15±3	0.150
Sex, male/female	23/27	22/9	0.028
Height, cm	158±13	166±14	0.015
Weight, kg	53.5±17.4	64.0±18.6	0.012
BMI, kg/m^2^	21.0±5.1	22.8±4.9	0.113
BMI z‐score	0.22±1.20	0.67±0.92	0.069
Heart rate, bpm	73±11	81±15	0.010
SBP, mmHg	107±13	137±17	<0.001
SBP z‐score	−0.21±0.97	2.2±1.5	<0.001
DBP, mmHg	62±11	71±16	0.004
DBP z‐score	−0.26±0.95	0.44±1.40	0.009
MAP, mmHg	76±10	90±15	<0.001
Antihypertensive drugs (n, %)		20, 64.5	<0.001

BMI indicates body mass index; DBP, diastolic blood pressure; MAP, mean arterial pressure; SBP, peripheral systolic blood pressure.

a Values are numbers, %, or means±SD.

b Values are adjusted for age and sex. Z‐scores represent the number of SDs from values in a reference population.^4^

### Peripheral and Central BP and Aortic PWV

As per definition, those with hypertension had significantly higher peripheral BP with systolic and diastolic BP 30±3 and 9±3 mmHg, respectively, greater in the hypertensive compared with normotensive groups, so that there was a more‐marked increase in pulsatility rather than mean BP. Central BP pressure components and PWV are summarized in Table 2. Average values of P1 were greater than those of P2 in normotensive and hypertensive groups so that augmentation pressure and AIx were negative. Both P1 and P2 were greater in hypertensive children compared with normotensive children, but the difference was proportionately greater for P1 than P2 so that augmentation pressure and AIx were similar in the 2 groups. Pulsatile components of central BP were all significantly greater in hypertensive compared with normotensive children, with the greatest difference being for PP1 (Table 2). Both P1 and P2 occurred earlier in systole in hypertensive compared with normotensive children with values of T1 and T2 15±6 and 16±5 ms lower, respectively, in hypertensive compared with normotensive children. The rate of initial pressure rise (PP1/T1) was therefore markedly greater in hypertensive compared with normotensive children (Table 2). Carotid‐femoral PWV was similar in both groups, but PWVss was higher in hypertensive compared with normotensive children (3.3±0.20 versus 2.5±0.16 m/s; *P*=0.004).

**Table 2:** Central Hemodynamics: Pressure Wave Morphology and Pulse Wave Velocity

Measure	Normotensive (n=50)	Hypertensive (n=31)^a^	*P* Value
Central systolic pressure points and timing			
P1, mmHg	90.0±1.7	111.6±2.0	<0.001
P2, mmHg	88.9±1.8	108.0±2.2	<0.001
Pes, mmHg	80.6±1.9	95.4±2.3	<0.001
T1, ms	120±4	105±5	0.041
T2, ms	225±3	209±4	0.005
Tes, ms	309±3	298±4	0.031
Central pulse pressures			
PP1, mmHg	28.0±1.7	39.7±2.1	<0.001
PP2, mmHg	26.9±1.5	37.1±1.8	<0.001
PPes, mmHg	18.7±1.1	24.5±1.3	0.001
Augmentation pressure and index			
AP, mmHg	−1.00±0.99	−2.6±1.2	0.322
AIx, %	−3.6±2.4	−4.1±3.0	0.913
Pulse wave velocities			
PWVss, m/s	2.5±0.16	3.3±0.20	0.004
PWVcf, m/s	5.9±0.18	5.9±0.19	0.802

AIx indicates augmentation index; AP, augmentation pressure; P1, blood pressure at the first systolic shoulder; P2, blood pressure at the second systolic shoulder; Pes, end‐systolic blood pressure; PP1, pulse pressure at P1; PP2, pulse pressure at P2; PPes, pulse pressure at Pes; PWVcf, carotid‐femoral pulse wave velocity; PWVss, pulse wave velocity by sum‐of‐squares; T1, timing of first systolic shoulder of central pressure: T2, timing of second systolic shoulder of central pressure; Tes, timing of the end‐systolic blood pressure.

a Values are adjusted for age and sex.

### LVOT Flow, SV, CO, and SVR

**Figure 2:**
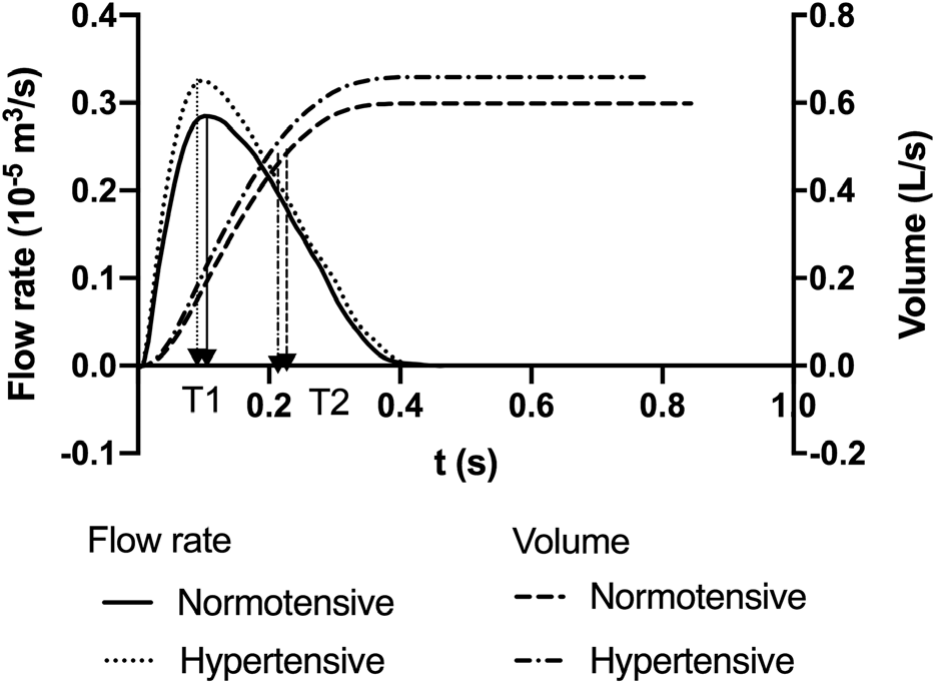
Average left ventricular flows and volumes in normotensive and hypertensive children.

Flow waveform characteristics are presented in Table 3. Compared with normotensive children, children with hypertension had significantly higher maximal flow velocity (U_max_) and mean flow velocity (U_mean_). Despite the earlier timing, T1 of P1 and U1, values of U1 were greater in hypertensive compared with normotensive children. Values of U2, V1, and V2 also tended to be greater in hypertensive compared with normotensive children (Figure 2) but the differences were not statistically significant. SVs calculated by integration of the flow waveform and from LV volumes were slightly greater in hypertensive children (3.2±5.3% and 3.5±5.5% greater for SV_flow_ and SV_vol_, respectively) compared with normotensive children, but the differences were not significant (Table 3). Because of both greater SV and HR (by 8±3 beats per minute) in hypertensive children, mean CO was 0.8±0.30 L/min (18.1±8.1%) greater in hypertensive compared with normotensive children. The percentage difference between CO in hypertensive compared with normotensive children was similar to that of MAP, so that SVR was similar in normotensive and hypertensive children (18.0±0.87 versus 19.3±1.1 mmHg/min/mL; *P*=0.374; Table 3).

**Table 3:** Central Hemodynamics: LVOT Flow Velocity, Flow, Stroke Volume, and Cardiac Output

Measure	Normotensive (n=50)	Hypertensive (n=31)^a^	*P* Value
LVOT flow velocity			
Umax, m/s	1.21±0.03	1.33±0.04	0.032
Umean, m/s	0.29±0.01	0.35±0.02	0.005
U1, m/s	1.13±0.03	1.26±0.04	0.010
U2, m/s	0.75±0.03	0.86±0.04	0.051
LVOT area and ejection volumes			
LVOT area, cm^2^	2.53±0.07	2.52±0.08	0.924
V1, mL	24.1±1.7	22.3±2.1	0.529
V2, mL	50.3±2.1	50.6±2.6	0.921
SV_flow_, mL	62.5±2.6	64.4±3.1	0.648
SV_vol_, mL	50.0±1.7	51.7±2.1	0.532
CO, L/min	4.5±0.23	5.30±0.27	0.029
SVR, mmHg/min/mL	18.00±0.87	19.3±1.1	0.374

CO indicates cardiac output; LVOT area, left ventricular outflow tract area; SV_flow_, stroke volume obtained by integration of LVOT flow; SVR, systemic vascular resistance; SV_Vol_, stroke volume obtained from ventricular dimensions; U1, flow velocity at T1; U2, flow velocity at T2; Umax, peak of flow velocity; Umean, mean of flow velocity; V1, ejection volume at T1; V2, ejection volume at T2.

a Values are adjusted for age and sex.

### Wave Separation and Intensity Analysis

**Figure 3:**
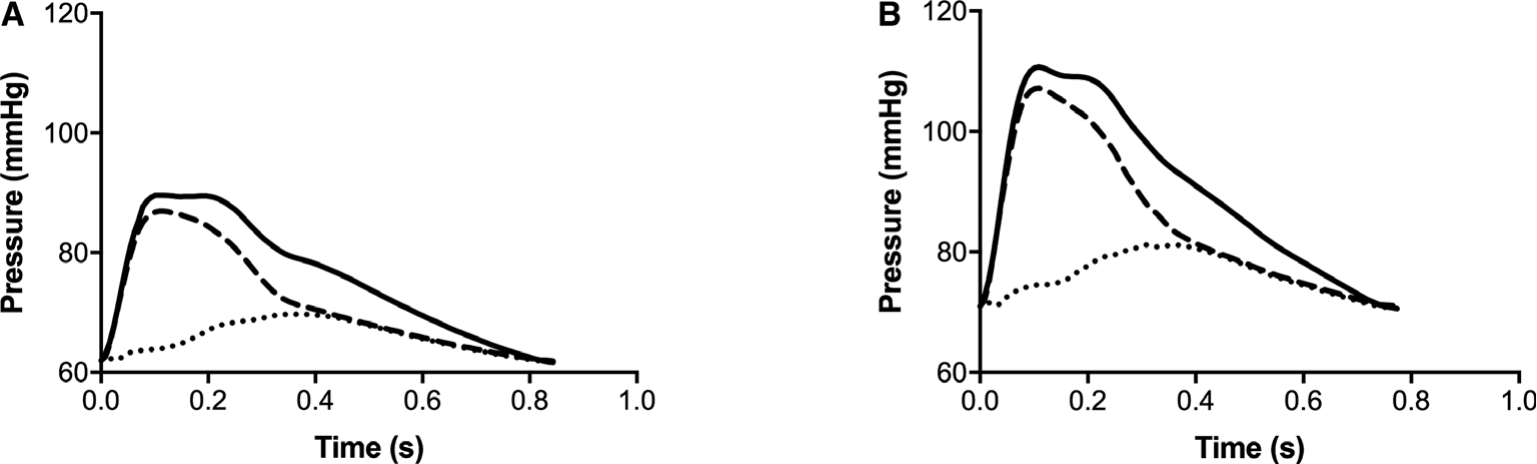
Average central pressure and corresponding decomposed forward and backward pressure waveforms in children. (**A**) Normotensive children and (**B**) hypertensive children. Solid lines show measured pressure, dashed lines show forward pressure, and dotted lines show backward pressure.

Average central pressure and the corresponding decomposed forward and backward pressure waveforms in normotensive and hypertensive children are presented in Figure 3. Peak amplitudes of the forward and backward pressure waves and of values of individual forward and backward components of pressure at characteristic points on the pressure waveform (P1, P2, and Pes) and timings of these are shown in Table 4. Both peak forward and backward amplitudes were greater in hypertensive compared with normotensive children. P1 and P2 were determined mainly by the forward wave in both hypertensive and normotensive children, and components of the forward wave for these values were greater in hypertensive compared with normotensive children. Reflection index, calculated as the ratio of the maximal amplitude of the backward to forward wave, was similar in hypertensive and normotensive children as were ratios of the backward to forward components of P1, P2, and Pes.

**Table 4:** Forward and Backward Pressure Wave Decomposition

Measurement	Normotensive (n=50)	Hypertensive (n=31)^a^	*P* Value
Amplitudes of pressure components			
Max Pf, mmHg	25.2±1.6	37.0±2.0	<0.001
Max Pb, mmHg	9.30±0.53	11.50±0.65	0.012
P1f, mmHg	24.7±1.6	36.1±1.9	<0.001
P1b, mmHg	3.30±0.36	3.60±0.45	0.632
P2f, mmHg	20.4±1.3	29.8±1.6	<0.001
P2b, mmHg	6.50±0.62	7.30±0.76	0.466
Pesf, mmHg	11.20±0.71	15.80±0.88	<0.001
Pesb, mmHg	6.70±0.56	7.50±0.69	0.338
Reflection index and backward/forward ratios			
Rindex	0.39±0.02	0.34±0.03	0.177
R_P1_	0.15±0.02	0.10±0.02	0.097
R_P2_	0.35±0.03	0.26±0.04	0.134
R_Pes_	0.73±0.10	0.50±0.13	0.160
Timing of pressure components			
Tarrival, ms	99±8	92±10	0.585
TmaxPf, ms	117±5	121±6	0.603
TmaxPb, ms	342±8	323±10	0.175

Max Pb indicates peak value (amplitude) of backward pressure wave; Max Pf, peak value (amplitude) of forward pressure wave; P1b, backward component of P1; P1f, forward component of P1; P2b, backward component of P2; P2f, forward component of P2; Pesb, backward component of end‐systolic pressure; Pesf, forward component of end‐systolic pressure; Rindex: reflection index; R_P1_, ratio of P1b to P1f; R_P2_, ratio of P2b to P2f; R_Pes_, ratio of Pesb to Pesf; Tarrival, arrival time of backward pressure wave; TmaxPb, timing of peak value of backward pressure wave.; TmaxPf, timing of peak value of forward pressure wave.

a Values are adjusted for age and sex.

Wave intensities and timing of wave intensity components are shown in Table 5. All wave intensity components, including amplitudes and area of forward and backward wave intensities except the amplitude of the backward expansion wave, were greater in hypertensive compared with normotensive children. Size of the forward expansion wave, indicative of a braking action of the left ventricle on forward pressure wave propagation, was particularly marked in the hypertensive group (Table 5). The peak of the backward expansion wave arrived earlier in hypertensive children (Table 5) but timings of other waves were not significantly different between normotensive and hypertensive children (Table 5).

**Table 5:** Wave Intensity Analysis

Measurement	Normotensive (n=50)	Hypertensive (n=31)^a^	*P* Value
Amplitudes of wave intensity components			
F_comp_, W/m^2^	91.5±10.2	166.3±12.6	<0.001
B_comp_, W/m^2^	6.0±1.2	11.6±1.5	0.006
F_exp_, W/m^2^	15.1±1.9	27.7±2.3	<0.001
B_exp_, W/m^2^	4.8±1.5	6.7±1.9	0.454
Areas of wave intensity components			
F_comp_ area, J/m^2^	3.50±0.35	6.10±0.42	<0.001
B_comp_ area, J/m^2^	0.22±0.03	0.34±0.03	0.004
F_exp_ area, J/m^2^	0.88±0.10	1.60±0.12	<0.001
B_exp_ area, J/m^2^	0.39±0.04	0.64±0.05	<0.001
Timing of wave intensity components (maximal amplitude)			
TF_comp_, ms	30±1	29±1	0.439
TB_comp_, ms	88±10	95±12	0.696
TF_exp_, ms	284±6	272±7	0.227
TB_exp_, ms	306±25	207±31	0.018

B_comp_ area indicates area of backward wave intensity; B_comp_, peak value (amplitude) of backward wave intensity; B_exp_ area, area of backward expansion wave intensity; B_exp_, peak value of backward expansion wave intensity; F_comp_ area, area of forward wave intensity; F_comp_, peak value (amplitude) of forward wave intensity; F_exp_ area, area of forward expansion wave intensity; F_exp_, peak value of forward expansion wave intensity; TB_comp_, timing of peak of backward wave intensity; TB_exp_, timing of peak of backward expansion wave intensity; TF_comp_, timing of peak of forward wave intensity; TF_exp_, timing of peak of forward expansion wave intensity.

a Values are adjusted for age and sex.

### Cardiac and Arterial Contributions to the Difference in Steady‐Sate and Pulsatile Components of BP Between Hypertensive and Normotensive Children

**Figure 4:**
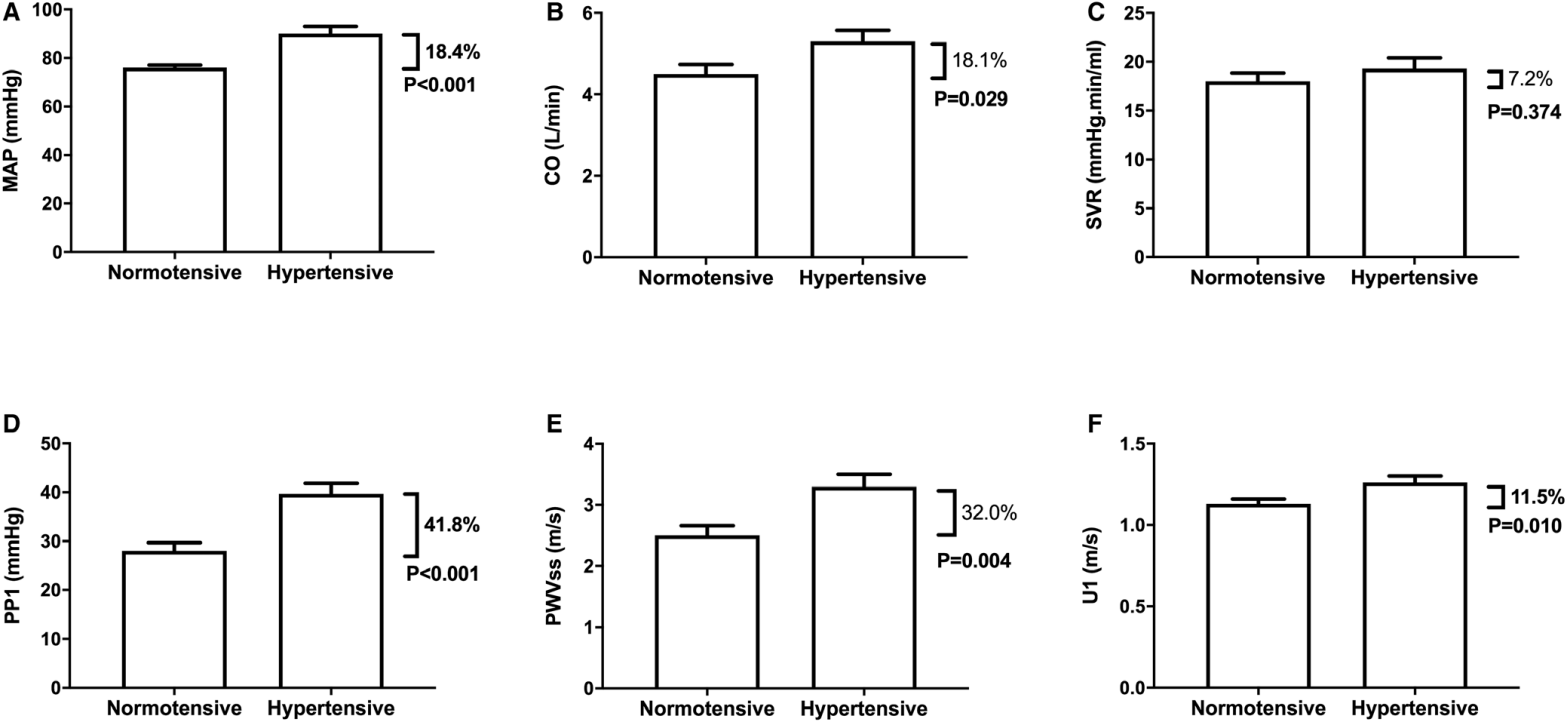
Major hemodynamic measures in normotensive (NT) and hypertensive (HT) children. (**A**) Mean arterial pressure (MAP), (**B**) cardiac output (CO), (**C**) systemic vascular resistance (SVR), (**D**) central pulsatile pressure at the first systolic shoulder (PP1), (**E**) proximal aortic pulse wave velocity (PWVss), and (**F**) left ventricular outflow velocity at the first systolic shoulder. Percentages refer to differences between normotensive and hypertensive children.

The greater MAP in hypertensive compared with normotensive children was explained by greater CO rather than SVR (Figure 4). Greater PP1 in hypertensive compared with normotensive children was explained by a combination of greater PWVss and greater early ejection velocities (Figure 4).

### Influence of Treatment and Adjustment for Height and Body Surface Area on Differences Between Hypertensive and Normotensive Children

Results were not materially altered when comparisons were made between untreated hypertensive and normotensive children (Tables S1 through S5). Differences between hypertensive and control groups in key hemodynamic measures (CO, SV, SVR, U1, PWVss, and carotid‐femoral PWV) were similar irrespective of whether the comparison was adjusted for age, sex, height, and body surface area and (in the case of PWV) MAP, HR, and LVOT cross‐sectional area (Table S6). In subsample analysis in boys alone, similar differences between hypertensive and control groups were noted, but not all differences that were significant in the whole sample reached statistical significance in the subsample because of the smaller sample size of the latter. Correction for body surface area, but not height, did attenuate the difference between CO in the hypertensive and normotensive groups, consistent with greater mass being a driver of increased CO.

## Discussion

To the best of our knowledge, this is the first comprehensive characterization of central hemodynamics in children with primary hypertension. There are 2 major findings. First, increased MAP, the steady‐state component of BP, in hypertension is attributable to an increase in HR and CO rather than increase in SVR. Second, increased pulsatility of BP in children with primary hypertension, which is more marked than the increase in MAP, is explained by a combination of increased proximal aortic stiffness (as measured by PWVss) and increased LV ejection velocity. These findings are consistent with, and extend previous studies in, adolescents and in young adults. Chirico et al^9^ measured CO by echocardiography in adolescents with primary hypertension and found elevated BP to be explained by increased CO rather than SVR. Arterial properties were not measured in the study by Chirico et al, but, in the ENIGMA population study on young adults, CO (by inert‐gas rebreathing) and PWV were measured and elevated BP was explained by increased SV and PWV rather than increased SVR.^10^ The present study extends the findings of the ENIGMA study with respect to proximal PWV as a determinant of pulsatility in primary hypertension in young adults to children and also identifies increased LV ejection velocity as an additional determinant of pulsatility in such children.

Measurement of flow velocity in addition to pressure allowed us to perform a comprehensive wave separation and intensity analysis, which confirmed that the main difference between hypertensive and normotensive children was an increase in the forward compression wave indicative of a hyperkinetic state. While backward wave components were also increased, this was likely secondary to the increase in the forward wave. Increased pressure wave “reflection,” usually inferred from pressure wave morphology (eg, from AIx, which is now recognized to be influenced by ventricular dynamics as well as by reflection), has been implicated in systolic hypertension in adults. However, in the present study, we found no evidence of increased reflection as measured by AIx or by the ratio of backward to forward wave components in hypertensive children.

When considering the primary hemodynamic alteration in children with hypertension, it is important to consider whether the increased proximal PWV we observed was a primary phenomenon or secondary to the increased MAP. Pressure dependence of PWV attributed to distension of the arterial wall transferring wall stress to stiffer elements within the wall is well recognized. In adults, acute modulation of transmural pressure (equivalent to an acute increase in MAP) or direct modulation of MAP using vasoactive drugs increases carotid‐femoral PWV and intrathoracic PWV (which theoretically is more closely related to PWVss) over a range of ≈0.5 to 1.0 m/s per 10 mmHg.^11^, ^12^ In the present study, the difference between PWVss in the hypertensive and control groups was 0.8 m/s and that for MAP 14 mmHg giving ratios of 0.6 m/s per 10 mmHg. Thus, the increase in PWV could be secondary to the increased MAP. Statistical adjustment for MAP did not alter the difference in proximal PWV between hypertensive and normotensive subjects, but this adjustment could have been limited by the relatively small number of subjects. The findings of the present study that primary hypertension in children is mainly a phenomenon arising from overactivity of the heart and proximal aorta is in stark contrast to the established view of primary hypertension as being a condition caused by increased resistance of the microvasculature. Given the tracking of BP from childhood to adulthood,^13^ this calls into question the focus on the microvasculature as the main cause of primary hypertension in adults.^14^, ^15^ It is possible that microvascular dysfunction occurs through remodeling secondary to increased MAP or pulsatility as proposed by Folkow.^14^, ^16^ Furthermore, it suggests that therapies, such as beta‐adrenergic antagonists, that reduce cardiac activity may be more effective in children than in adults.

Our study did not address the underlying etiology of the hemodynamic determinants of hypertension. However, increased sympathetic activity has been suggested as the underlying cause for primary hypertension in children, particularly when hypertension is associated with obesity.^17^ Sympathetic activity was not measured in the present study, but increased sympathetic activity would be entirely consistent with the increased HR and increased ventricular ejection velocities we observed. It could also contribute to the elevated proximal PWV independent of effects of MAP. In the present study, body mass index was similar in hypertensive and normotensive groups, and increased sympathetic activity could be an important cause of primary hypertension in nonobese children. Renal sodium handling is likely to be another important cause of the difference between hypertensive and normotensive children. The current study did not directly measure sympathetic activity nor address renal sodium handling and, as such, does not fully characterize what is likely to be a complex and heterogeneous phenotype.

There are a number of other important limitations to our study. We studied a relatively small number of hypertensive children and were not able to meaningfully stratify our analysis by age and sex. Further studies are required to characterize hypertension in different age groups and according to other characteristics. Many of our hypertensive children were on some antihypertensive treatment, and although adjustment for treatment or analysis in untreated children made no difference to our conclusions, further studies on a larger group of untreated children are indicated. Noninvasive measurements of hemodynamics are inevitably subject to experimental error and measurements of peripheral and central BP are subject to calibration error. Our method of calibration of carotid waveforms by MAP derived from radial tonometry and brachial and diastolic BP is subject to error attributable to brachial to radial amplification.^18^ While these errors may have influenced absolute values of BP, they are unlikely to have influenced values in the hypertensive relative to the normotensive group, which was the main focus of the present study.

In conclusion, the present study suggests that primary hypertension in children results from cardiac overactivity characterized by increased HR and LV ejection velocities and increased proximal aortic stiffness.

## Sources of Funding

This work was supported by a British Heart Foundation Project grant PG/17/50/32903. The study also received support from the National Institute for Health Research Clinical Research Facility and Biomedical Research Centre based at Guy’s and St. Thomas’ NHS Foundation Trust and King’s College London.

## Disclosures

None.

## Supporting information


**Table S1.** Characteristics of Normotensive and Hypertensive Children: Comparison of Values in Untreated Hypertensive and Normotensive Children
**Table S2.** Central Hemodynamics: Pressure Wave Morphology and Pulse Wave Velocity: Comparison of Values in Untreated Hypertensive and Normotensive Children
**Table S3.** Central Hemodynamics: LVOT Flow Velocity, Flow, Stroke Volume, and Cardiac Output: Comparison of Values in Untreated Hypertensive and Normotensive Children
**Table S4.** Forward and Backward Pressure Wave Decomposition: Comparison of Values in Untreated Hypertensive and Normotensive Children
**Table S5.** Wave Intensity Analysis: Comparison of Values in Untreated Hypertensive and Normotensive Children
**Table S6.** Influence of Adjustment for Age, Sex, Height, Body Surface Area, and Other Factors on Key Hemodynamic Measures
