# Supplementary material for: Hemodynamic Characterization of Primary Hypertension in Children and Adolescents
Source: J Am Heart Assoc. 2020 Jun 6;9(12):e015097. doi: 10.1161/JAHA.119.015097 (PMC7429040; doi:10.1161/JAHA.119.015097)
Supplement: Supplementary file 1 — Table S1. Characteristics of Normotensive and Hypertensive Children: Comparison of Values in Untreated Hypertensive and Normotensive Children Table S2. Central Hemodynamics: Pressure Wave Morphology and Pulse Wave Velocity: Comparison of Values in Untreated Hypertensive and Normotensive Children Table S3. Central Hemodynamics: LVOT Flow Velocity, Flow, Stroke Volume, and Cardiac Output: Comparison of Values in Untreated Hypertensive and Normotensive Children Table S4. Forward and Backward Pressure Wave Decomposition: Comparison of Values in Untreated Hypertensive and Normotensive Children Table S5. Wave Intensity Analysis: Comparison of Values in Untreated Hypertensive and Normotensive Children Table S6. Influence of Adjustment for Age, Sex, Height, Body Surface Area, and Other Factors on Key Hemodynamic Measures [file JAH3-9-e015097-s001.pdf]

SUPPLEMENTAL MATERIAL

Table S1. Characteristics of normotensive and hypertensive children: comparison of values in untreated hypertensive and normotensive children.

Characteristic*	Normotensive (n=50)	Hypertensive (n=13)	P value
Age (years)	14±3	14±3	0.616
Sex, male/female	23/27	6/7	0.992
Height (cm)	160±13	163±13	0.723
Weight (kg)	53.5±17.4	59.1±16.2	0.352
BMI (kg/m^2)	21.0±5.1	22.7±5.3	0.334
BMI z-score	0.22±1.2	0.76±0.90	0.115
Heart rate (bpm)	73±11	83±18	0.011
SBP (mmHg)	107±13	133±19	<0.001
SBP z-score	-0.21±0.97	2.1±1.8	<0.001
DBP (mmHg)	62±11	72±13	0.003
DBP z-score	-0.26±0.95	0.66±1.4	0.004
MAP (mmHg)	76±10	91±15	<0.001

BMI, body mass index; SBP, peripheral systolic blood pressure; DBP, diastolic blood pressure; MAP, mean arterial pressure. *Values are numbers, % or means±SD.

Table S2. Central hemodynamics: pressure wave morphology and pulse wave velocity: comparison of values in untreated hypertensive and normotensive children.

Measure	Normotensive (n=50)	Hypertensive* (n=13)	P value
**Central systolic pressure points and timing**			
P1 (mmHg)	89.8±1.4	110.0±2.6	<0.001
P2 (mmHg)	89.0±1.6	109.3±3.0	<0.001
Pes (mmHg)	80.8±1.6	96.3±3.0	<0.001
T1 (ms)	120±4	103±8	0.085
T2 (ms)	224±3	209±6	0.035
Tes (ms)	309±3	303±6	0.452
**Central pulse pressures**			
PP1 (mmHg)	27.7±1.4	38.0±2.7	0.001
PP2 (mmHg)	26.9±1.4	37.3±2.7	0.001
PPes (mmHg)	18.7±1.1	24.3±2.0	0.017
**Augmentation pressure and index**			
AP (mmHg)	-0.79±0.82	-0.68±1.5	0.952
Aix (%)	-3.0±2.2	-2.5±4.2	0.914
**Pulse wave velocities**			
PWVss (m/s)	2.5±0.14	3.2±0.27	0.029
PWVcf (m/s)	5.8±0.14	5.4±0.26	0.217

P1, blood pressure at the first systolic shoulder; P2, blood pressure at the second systolic shoulder; Pes, end-systolic blood pressure; T1, timing of first systolic shoulder of central pressure: T2, timing of second systolic shoulder of central pressure; Tes, timing of the end-systolic blood pressure; PP1, pulse pressure at P1; PP2, pulse pressure at P2; PPes, pulse pressure at Pes; AP, augmentation pressure; Aix, augmentation index; PWVss, pulse wave velocity by sum-of-squares; PWVcf, carotid-femoral pulse wave velocity. *Values are adjusted for age and sex.

Table S3. Central hemodynamics: LVOT flow velocity, flow, stroke volume and cardiac output: comparison of values in untreated hypertensive and normotensive children.

Measure	Normotensive (n=50)	Hypertensive* (n=13)	P value
**LVOT flow velocity**			
Umax (m/s)	1.21±0.03	1.36±0.05	0.015
Umean (m/s)	0.30±0.01	0.38±0.02	<0.001
U1 (m/s)	1.13±0.03	1.30±0.05	0.005
U2 (m/s)	0.76±0.03	0.96±0.06	0.007
**LVOT area and ejection volumes**			
LVOT area (cm^2)	2.45±0.06	2.25±0.12	0.145
V1 (ml)	23.4±1.4	18.7±2.6	0.118
V2 (ml)	48.6±1.8	46.4±3.3	0.547
SV_{flow} (ml)	60.6±2.3	61.7±4.3	0.827
SV_{vol} (ml)	48.4±1.7	47.7±3.3	0.859
CO (L/min)	4.4±0.17	5.0±0.32	0.079
SVR(mmHg.min/ml)	18.4±0.70	18.9±1.3	0.732

Umax, peak of flow velocity; Umean, mean of flow velocity; U1, flow velocity at T1; U2, flow velocity at T2; LVOT area, left ventricular outflow tract area; V1, ejection volume at T1; V2, ejection volume at T2; SV_{flow}, stroke volume obtained by integration of LVOT flow; SV_{Vol}, stroke volume obtained from ventricular dimensions; CO, cardiac output; SVR, systemic vascular resistance. *Values are adjusted for age and sex.

Table S4. Forward and backward pressure wave decomposition: comparison of values in untreated hypertensive and normotensive children.

Measurement	Normotensive (n=50)	Hypertensive* (n=13)	P value
**Amplitudes of pressure components**			
Max Pf (mmHg)	25.1±1.4	36.4±2.6	<0.001
Max Pb (mmHg)	9.3±0.54	11.2±1.0	0.111
P1f (mmHg)	24.5±1.4	35.3±2.6	0.001
P1b (mmHg)	3.2±0.31	2.7±0.59	0.462
P2f (mmHg)	20.5±1.2	31.0±2.3	<0.001
P2b (mmHg)	6.4±0.60	6.3±1.1	0.920
Pesf (mmHg)	11.2±0.72	16.3±1.4	0.001
Pesb (mmHg)	6.7±0.55	6.8±1.0	0.922
**Reflection index and backward/forward ratios**			
Rindex	0.39±0.02	0.32±0.05	0.204
R_{P1}	0.14±0.02	0.09±0.03	0.134
R_{P2}	0.34±0.04	0.22±0.07	0.128
R_{Pes}	0.74±0.11	0.41±0.21	0.186
**Timing of pressure components**			
Tarrival (ms)	101±8	102±16	0.940
TmaxPf (ms)	118±5	124±9	0.523
TmaxPb (ms)	342±8	353±15	0.531

Max Pf, peak value (amplitude) of forward pressure wave; Max Pb, peak value (amplitude) of backward pressure wave; P1f, forward component of P1; P1b, backward component of P1; P2f, forward component of P2; P2b, backward component of P2; Pesf, forward component of end-systolic pressure; Pesb, backward component of end-systolic pressure; Rindex: reflection index; R_{P1}, ratio of P1b to P1f; R_{P2}, ratio of P2b to P2f; R_{Pes}, ratio of Pesb to Pesf; Tarrival, arrival time of backward pressure wave; TmaxPf, timing of peak value of forward pressure wave; TmaxPb, timing of peak value of backward pressure wave. *Values are adjusted for age and sex.

Table S5. Wave intensity analysis: comparison of values in untreated hypertensive and normotensive children.

Measurement	Normotensive (n=50)	Hypertensive* (n=13)	P value
**Amplitudes of wave intensity components**			
F_{comp} (W/m^2)	92.8±9.1	168.1±17.2	<0.001
B_{comp} (W/m^2)	6.0±1.1	11.6±2.0	0.016
F_{exp} (W/m^2)	15.7±1.8	28.6±3.5	0.002
B_{exp} (W/m^2)	5.0±1.6	7.4±2.9	0.469
**Areas of wave intensity components**			
F_{comp} area (J/m^2)	3.6±0.30	6.1±0.57	<0.001
B_{comp} area (J/m^2)	0.22±0.03	0.36±0.05	0.009
F_{exp} area (J/m^2)	0.89±0.09	1.7±0.17	<0.001
B_{exp} area (J/m^2)	0.40±0.04	0.70±0.07	<0.001
**Timing of wave intensity components (maximal amplitude)**			
TF_{comp} (ms)	30±1	29±2	0.814
TB_{comp} (ms)	87±10	117±19	0.177
TF_{exp} (ms)	284±5	293±10	0.420
TB_{exp} (ms)	300±27	211±50	0.122

F_{comp}, peak value (amplitude) of forward wave intensity; B_{comp}, peak value (amplitude) of backward wave intensity; F_{exp}, peak value of forward expansion wave intensity; B_{exp}, peak value of backward expansion wave intensity; F_{comp} area, area of forward wave intensity; B_{comp} area, area of backward wave intensity; F_{exp} area, area of forward expansion wave intensity; B_{exp} area, area of backward expansion wave intensity; TF_{comp}, timing of peak of forward wave intensity; TB_{comp}, timing of peak of backward wave intensity; TF_{exp}, timing of peak of forward expansion wave intensity; TB_{exp}, timing of peak of backward expansion wave intensity. *Values are adjusted for age and sex.

Table S6. Influence of adjustment for age, sex, height, body surface area and other factors on key haemodynamic measures.

	Normotensive (n=50)	Hypertensive (n=31)	Difference (%)	P value
**CO (L/min)**				
Unadjusted	4.3±0.18	5.4±0.36	25.6	0.004
Age + Sex	4.5±0.23	5.3±0.27	17.8	0.029
Boys only + Age	4.6±0.40	5.5±0.38	19.6	0.125
Age + Sex + Height	4.5±0.23	5.3±0.28	17.8	0.036
Age + Sex + BSA	4.6±0.21	5.1±0.25	10.9	0.087
**CI (L/min/m^2)**				
Unadjusted	2.9±0.91	3.2±1.7	10.3	0.073
Age + Sex	2.8±1.2	3.2±1.4	14.3	0.071
**SV_{flow} (ml)**				
Unadjusted	60.4±2.6	67.5±3.7	11.8	0.108
Age + Sex	62.5±2.6	64.4±3.1	3.0	0.648
Boys only + Age	68.2±4.8	69.5±4.5	1.9	0.838
Age + Sex + Height	62.7±2.6	64.2±3.1	2.4	0.713
Age + Sex + BSA	63.7±2.3	62.8±2.8	-1.4	0.826
**SVR (mmHg.min/ml)**				
Unadjusted	18.4±0.69	18.7±1.3	1.6	0.835
Age + Sex	18.0±0.87	19.3±1.1	7.2	0.374
Boys only + Age	17.4±1.4	17.4±1.3	0	0.998
Age + Sex + Height	18.0±0.88	19.3±1.1	7.2	0.347
Age + Sex + BSA	17.8±0.85	19.6±1.0	10.1	0.204
**U1 (m/s)**				
Unadjusted	1.13±0.03	1.26±0.04	11.5	0.008
Age + Sex	1.13±0.03	1.26±0.04	11.5	0.010
Boys only + Age	1.11±0.05	1.23±0.05	10.8	0.101
Age + Sex + Height	1.13±0.03	1.26±0.04	11.5	0.009
**PWVss (m/s)**				
Unadjusted	2.5±0.12	3.4±0.25	36.0	0.001
Age + Sex	2.5±0.16	3.3±0.20	32.0	0.004
Boys only + Age	2.8±0.28	3.3±0.27	17.9	0.253
Age + Sex + MAP + HR	2.5±0.17	3.3±0.22	32.0	0.013
Age + Sex + MAP + HR + LVOT area	2.5±0.17	3.3±0.22	32.0	0.014
**PWVcf (m/s)**				
Unadjusted	5.7±0.17	6.1±0.22	7.0	0.145
Age + Sex	5.9±0.18	5.9±0.19	0	0.802

Boys only + Age	6.0±0.24	6.1±0.20	1.7	0.948
Age + Sex + MAP + HR	6.0±0.22	5.8±0.23	-3.3	0.691
Age + Sex + MAP + HR + LVOT area	5.9±0.20	5.9±0.21	0	0.823

BSA, body surface area; CO, cardiac output; CI, cardiac index; SV, stroke volume; SVR, systemic vascular resistance; PWVss, pulse wave velocity by sum-of-squares; PWVcf, carotid-femoral pulse wave velocity; LVOT, left ventricular outflow tract.* Adjusted for age.
